# Self-reported and pill count measures of adherence to oral HIV PrEP among female sex workers living in South-Western Uganda

**DOI:** 10.1371/journal.pone.0277226

**Published:** 2022-11-10

**Authors:** Lydia Jacenta Nakiganda, Andrew E. Grulich, Isobel Mary Poynten, David Serwadda, Jeremiah Mulamba Bazaale, Jeff Jin, Benjamin R. Bavinton

**Affiliations:** 1 Kirby Institute for Infection and Immunity in Society, UNSW Sydney, Kensington, Australia; 2 Rakai Health Sciences Program, Kalisizo, Uganda; 3 Makerere University School of Public Health, Kampala, Uganda; University of Washington, UNITED STATES

## Abstract

**Background:**

Female sex workers (FSWs) in Uganda are at high risk of HIV infection. Scaling up oral pre-exposure prophylaxis (PrEP) will reduce HIV incidence if high levels of adherence are maintained. This study evaluates PrEP adherence using clinic-based pill counts and self-reported measures, and factors associated with protective levels of adherence.

**Methods:**

Participants were sex workers who had been taking PrEP for at least 5 months and were attending routine follow-up visits for PrEP care in fishing communities and along the Trans-African Highway. Participants who had a pill count showing at least 85% use since their last clinic visit and those who reported taking their PrEP every day in the last 5 months were categorised as having ‘protective adherence’. Spearman’s correlation and weighted kappa assessed the relationship between pill count and self-reported measures. Bivariate and multivariate logistic regression was used to determine factors associated with protective adherence as measured by pill count.

**Results:**

We recruited 524 FSWs, with a median age of 29 years (IQR 23–35). Participants were recruited from fishing communities and Trans-African Highway towns (n = 297, 56.7%, and n = 227, 43.0%). Nearly three quarters (n = 372, 71.0%) of women were estimated to have protective adherence based on pill count (i.e., a pill count of >85%) and 50.4% by self-report in last 3 months. There was a strong positive association between self-reported measures and pill count measures (*r*_*est*_ = 0.6453, 95% CI = 0.5924–0.6927) and a moderate agreement between self-reported measures and pill count measures, κ = 0.544 (95%CI = 0.4869–0.6011, *p* < 0.001).

Factors associated with protective adherence included being aged 35 years or older (aOR = 2.40, 95% CI = 1.17–4.86), living in a fishing community (aOR = 1.45, 95% CI = 0.62–3.38), and having an STI in last 3 months (aOR = 1.64, 95% CI = 1.07–2.49).

**Conclusion:**

Our findings indicate that PrEP-experienced FSWs attending clinical follow-up visits reported high protective levels of oral pre-exposure prophylaxis, as measured by both pill count and self-reported measures, and a moderate agreement between pill count and self-reported measures.

## Introduction

Female sex workers (FSWs) are over 13 times more likely to acquire HIV than all women of reproductive age in low- and middle-income countries [1], and contribute 18% of new HIV infections in Sub-Saharan Africa [2].Recent estimates from major cities in Eastern Africa include HIV prevalence of 75% in Kisumu, Kenya [3], 29.5% in Nairobi, Kenya [4], 15% in Dar es Salaam, Tanzania [5], and 33% in Kampala, Uganda [6].

Oral pre-exposure prophylaxis (PrEP) with tenofovir disoproxil fumarate/emtricitabine is highly effective in preventing HIV acquisition. PrEP use is recommended in populations at high risk by the World Health Organization [7,8] and PrEP demonstration and implementation studies targeting sex workers have been conducted in several Sub-Saharan African countries [9–14]. In Uganda, PrEP implementation projects were launched in 2017 [15]. A range of clinical trials and implementation and demonstration projects are also ongoing [16], mostly involving HIV serodiscordant couples [17–22]. Other populations involved in these studies are adolescents and young women [12,23], female sex workers [12,24], and populations experiencing substantial HIV risk including fisherfolk, truck drivers and men who have sex with men [25–27].

In Uganda, sex work is illegal [28]. Female sex work is highly concentrated in HIV-hyperendemic fishing communities along Lake Victoria and towns on the Trans-African Highway. In these settings, there is an urban culture characterised by high alcohol consumption and drug use, entertainment venues and other social and structural drivers of HIV including unemployment, high levels of poverty, population mobility and disconnection from families. The fishing industry also attracts a considerable influx of high-risk populations during peak fishing months [29,30] due to the high earning power and spending patterns of fishermen.

The key challenge in effective PrEP roll-out is achieving high levels of adherence. In heterosexual women, adherence to at least 6 TDF/FTC pills per week is required to provide HIV protection in the lower genital tract [31]. Accurately measuring adherence to PrEP remains a challenge [32,33]. The most accurate methods–drug concentrations in biological specimens–are expensive and not feasible in many resource-limited settings. Thus, inexpensive, simple methods like self-report, although subjective and susceptible to bias, may be a pragmatic option. In rural Uganda, there have been no studies reported comparing PrEP adherence using clinic-based pill counts and self-reported measures among FSWs on routine PrEP care.

Studying PrEP use among women attending routine care for a PrEP refill may highlight features that are associated with being able to stay on PrEP for longer periods of time. We evaluated PrEP adherence among FSWs attending routine PrEP care at Ugandan health clinics located in fishing communities on Lake Victoria and along the Trans-African Highway, investigated factors associated with protective levels of adherence and measured the agreement/concordance between two measures of adherence (self-reported vs pills count since last refill).

## Methods

### PrEP program

The PrEP program in clinics in Southwestern Uganda has been described in detail [12]. Among the high-risk areas designated for priority PrEP roll-out were HIV hyperendemic fishing communities on Lake Victoria and highway trading towns. Through community-wide mobilization led by health workers, peer leaders and village health teams, individuals are referred to health units for screening. These follow a risk assessment tool or the HIV consolidated guidelines designed by the Ugandan Ministry of Health [34]. Any individual deemed at substantial risk is invited to enroll at either a health unit or a designated outreach site which all have counsellors, laboratory technicians and clinicians. In the first year of receiving PrEP, clients are usually asked to return to clinics at one, three, six, nine and twelve months for refills, adherence counselling and screening for sexually transmitted infection (STIs) symptoms. However, refill schedules are flexible and are usually made according to client preference. Peer leaders, village health teams and health workers support client retention in the program.

### Study design and participants

A cross sectional study of FSWs was conducted between January to July 2021. We recruited FSWs in four fishing communities along Lake Victoria and in three Trans-African Highway towns who were attending routine PrEP follow-up visits at six health centres and one outreach site. Health workers were briefed about the study, and they identified potential participants from PrEP client registers. At some clinics, peer leaders also identified potential participants. Usually, these registers included data on last date of PrEP refill, duration on PrEP, telephone contacts, and home address. Eligible FSWs were required to: be aged ≥18 years; have serologically confirmed HIV-negative status on their most recent PrEP refill cards, have traded sex or exchanged sex for money or gifts in the 3 months preceding the interview, have been dispensed PrEP at the clinic for at least 5 months, and be recorded as taking PrEP at their last clinic visit. Potential participants were contacted directly by research team members via telephone or via peer leaders prior to the time of their visit. They were reminded of the date of their refill days and asked to visit their health centres or outreach sites on specific dates. Prospective participants attended a group information session about the study and participant information sheets were distributed. As clinic refill days are busy days, interviews were scheduled for subsequent days of their choice at their preferred nearest location. Surveys were administered during a one-on-one interview in Luganda or English (lasting 60–90 minutes), and responses were recorded on a paper-based questionnaire by the interviewer.

### Measures

The primary outcome of this analysis was adherence sufficiently high to be protective, hereafter referred to as ‘protective adherence’. This was measured in two ways. First, self-reported pill-taking in the previous three months was assessed with the question, “Thinking about the last 3 months, how often did you take all your PrEP pills?”, with the response options, "All my pills everyday", "Most of my pills", "About one half of my pills", "Very few of my pills" and "None of my pills". “All my pills” were categorised as 6–7 pills a week, “most of my pills” as 5 pills a week, “About one half of my pills” as 3–4 pills a week, “Very few of my pills” as 1–2 pills a week and “None of my pills” as no pills taken all week. Participants who reported "All my pills everyday" were categorised as having protective adherence, based on the need for ≥85% PrEP adherence (31).

Second, we used a pill count measure as the proportion of daily pills dispensed since the last visit that were taken, based on a count of remaining pills conducted by the study interviewers. We assumed the difference between the number of pills dispensed at the previous visit and the number of pills returned at the current visit represented the number of pills used and divided this by the number of days that had elapsed between the previous and current visits. Participants were defined as having protective adherence if their pill count was at least 85%—that is expected if they were taking PrEP daily. No biological measures of adherence were made.

Demographic data were collected including age, marital status, education, religion and area of residence. Area of residence was divided into two types: fishing communities and Trans-African Highway towns. A fishing community was defined as people living on the Lake Victoria shore or in a trading centre adjacent to a lake landing site where fishing is the primary economic activity [35]. Trans-African Highway towns included the densely populated towns on the Kigali-Kampala highway. Sexual behaviour data collected included number of sexual partners, sexual partner types, sex work, condom use, STIs, and alcohol and other drug use with partners before sex. Sexual partners were classified into distinct types: husband/steady partner (a partner to whom the participant was in a close, long-term, ongoing relationship with demonstrated affection, social support, and commitment); regular non-paying partners (a partner with whom the participant had sex with regularly or more than once but who did not pay money or goods in exchange for sex); regular paying partners (a partner with whom the participant had sex with regularly or more than once and who paid money or goods in exchange for sex); and casual paying partners (a first-time or one-time partner whom the participant received money or goods in exchange for sex). We also asked if the participant had had any STIs in the last 3 months, with the response options, “Yes” or “No”. Participants’ attitudes to PrEP were also collected and measured by a 5-point Likert scale. Symptoms of depression were assessed with the Patient Health Questionnaire (PHQ-9) [36]; a score of ≥10 was defined as moderate-severe depression.

#### Ethics statement

Participants who were willing to participate in the study gave written consent to participate in the study. Ethics approval was obtained from the Research and Ethics Committee of The AIDS Support Organisation (TASOREC/036/2020-UG-REC-009), the Ugandan Council of Science and Technology (HS813ES), and the University of New South Wales Human Research Ethics Committee (HC200357).

#### Statistical analysis

For baseline characteristics, the sample was divided into two groups: those who lived in the fishing communities and those who were living along the Trans-Africa Highway. Descriptive statistics (absolute and relative frequencies, means, standard deviation and quartiles etc) were conducted, and we used the Pearson’s Chi square test for comparisons. In addition, we aimed to compare self-reported adherence measures with clinic-based pill count measures of adherence and determine factors that predicted protective adherence by pill count. To compare the two types of adherence measures used, we grouped adherence percentage levels by pill count into five categories. A Spearman’s correlation (*r*_*s*_) test was performed to assess the relationship between these two adherence measures. In addition, weighted kappa statistics was used to assess the agreement between the two adherence measures [37]. We calculated weighted kappa with 95% confidence intervals (CI), using the following interpretation categories: No kappa agreement, <0; slight, 0.00–0.20; fair, 0.21–0.40; moderate, 0.41–0.60; substantial agreement, 0.61–0.80; and almost perfect, 0.81–1.00 [37].

We used bivariate and multivariable logistic regression models to determine associations between protective adherence to PrEP by pill count and participant characteristics. We report odds ratios (OR), adjusted odds ratios (aOR), 95% confidence intervals (CIs) and p-values for these associations. Factors with a p-value of <0.1 in the bivariable models were block entered into the multivariable model. Analyses were conducted using Stata version 17 (Stata Corp, College Station, TX).

## Results

Between January and July 2021, 524 participants were recruited, with a median age of 29 years (IQR 23–35). As shown in Table 1, participants were recruited from fishing communities and Trans-African Highway towns (n = 297, 56.7%, and n = 227, 43.3% respectively). Over half the participants had primary school level of education or less (n = 311, 59.4%), had other sources of income apart from sex work (n = 283, 54.0%), and were paid between 5,000 to 10,000 Ugandan Shillings (about USD $1.40 to $2.60) per client for their sex work (n = 296, 56.5%). Half of the women had husbands/steady partners (n = 262, 50.0%), one in five had regular non-paying partners (n = 114, 21.8%), over three-quarters had regular paying partners (n = 404, 77.1%) and almost all had casual paying partners (n = 510, 97.3%). Most of the women had been taking PrEP for 1 to 2 years (n = 395, 75.4%,).

**Table 1:** Characteristics of female sex worker study participants (n = 524).

Characteristics	Trans Africa Highway (n = 227)	Fishing community (n = 297)	Total n (%)	P-Value	P-trend
**Age, years**					
18–24	70 (30.8)	92 (30.9)	162 (30.9)		0.557
25–34	113 (49.8)	157 (52.9)	270 (51.5)		
35+	44 (19.4)	48 (16.2)	92 (17.6)		
**Education**					
Primary or less	126 (55.5)	185 (62.3)	311 (59.4)	0.119	
Secondary or more	101 (44.5)	112 (37.7)	213 (40.7)		
**Marital status**					
Single/never married	61 (26.9)	60 (20.2)	121 (23.1)	0.049	
Married	14 (6.2)	33 (11.1)	47 (9.0)		
Divorced/separated/widowed	152 (67)	204 (68.7)	356 (68.0)		
**Source of income**					
Sex work only	126 (55.5)	115 (38.7)	241 (46.0)	<0.001	
Sex work and any other occupation	101 (44.5)	182 (61.3)	283 (54.0)		
**Money paid per client (Ugandan Shillings)**					
≤5000 (1.4 USD)	9 (4)	5 (1.7)	14 (2.7)		0.022
5000–10000 (1.4 USD– 2.8 USD)	114 (50.2)	182 (61.3)	296 (56.5)		
≥ 10000 (≥ 2.8 USD)	104 (45.8)	110 (37.0)	214 (40.9)		
**Religion**					
Catholic	89 (39.2)	158 (53.2)	247 (47.1)		
Protestant	77 (33.9)	68 (22.9)	145 (27.7)	0.003	
Saved/Pentecostal	13 (5.7)	22 (7.4)	35 (6.7)		
Muslim	45 (19.8)	49 (16.5)	94 (17.9)		
Others	3 (1.3)	0	3 (0.6)		
**No. of male sexual partners in last 3 months**					
Less than 100 men	21 (9.3)	58 (19.5)	79 (15.1)	0.001	
More than or equal to 100 men	206 (90.8)	239 (80.5)	445 (84.9)		
**Years since PrEP dispensed**					
< 1 year	52 (22.9)	22 (7.4)	74 (14.1)		<0.001
1–2 years	171 (75.3)	224 (75.4)	395 (75.4)		
2–3 years	4 (1.8)	51 (17.2)	55 (10.5)		
**Pills taken in the past 7 days**					
All my pills everyday	139 (61.2)	228 (76.8)	367 (70.0)		<0.001
Most of my pills	56 (24.7)	42 (14.1)	98 (18.7)		
About one half of my pills	10 (4.4)	0 (0)	10 (1.91)		
Very few of my pills	12 (5.3)	9 (3.0)	21 (4.0)		
None of my pills	10 (4.41)	18 (6.1)	28 (5.34)		
**Pills taken in the past 3 months**					
All my pills everyday	98 (43.2)	166 (55.9)	264 (50.4)		0.001
Most of my pills	92 (40.5)	98 (33.0)	190 (36.3)		
About one half of my pills	19 (8.4)	7 (2.4)	26 (4.9)		
Very few of my pills	17 (7.5)	19 (6.4)	36 (6.9)		
None of my pills	1 (0.4)	7 (2.4)	8 (1.5)		
**Husband/steady partner in last 3 months**					
Yes	97 (42.7)	165 (55.6)	262 (50.0)	0.004	
No	130 (57.3)	132 (44.4)	262 (50.0)		
**Regular paying partners in last 3 months**					
Yes	162 (71.4)	242 (81.5)	404 (77.1)	0.006	
No	65 (28.6)	55(18.5)	120 (22.9)		
**Regular non-paying regular partners in last 3 months**					
Yes	43 (18.9)	71 (23.9)	114 (21.8)	0.172	
No	184 (81.1)	226 (76.1)	410 (78.2)		
**Casual paying partners in last 3 months**					
Yes	221 (97.4)	289 (97.3)	510 (97.3)	0.972	
No	6 (2.6)	8 (2.7)	14 (2.7)		
**Intent to continue taking PrEP in next 12 months**					
Yes	207 (91.2)	287 (96.6)	494 (94.3)	0.008	
No	20 (8.8)	10 (3.4)	30 (5.7)		
**STI in last 3 months**					
Yes	129 (56.8)	108 (36.4)	237 (45.2)	<0.001	
No	98 (43.2)	189 (63.6)	287 (54.8)		
**Condom use at last sex with regular partners**					
Yes	77 (47.53)	140 (57.9)	217 (53.7)	0.04	
No	85 (52.5)	102 (42.2)	187 (46.3)		
**Condom use at last sex with casual partners**					
Yes	125 (56.6)	177 (61.3)	302 (59.2)	0.286	
No	96 (43.4)	112 (38.7)	208 (40.8)		

P-trend***.

For self-reported adherence in the last 3 months (Table 2), 264 (50.4%) women reported that they took all of their pills, 190 (36.3%) most of their pills, 26 (4.9%) about one half of their pills, 36 (6.9%) very few of their pills, and 8 (1.5%) none of their pills. For the pill count adherence measures (Table 2), 372 (70.%) had pill count of ≥85%, 56 (10.7%) of 70–84%, 34 (6.5%) of 55–69%, 22 (4.2%) of 41–55%, and 40 (7.6%) women had a proportion of daily pills taken of 0–40%. There was a strong positive association between self-reported measures and proportion of daily pills taken by pill count measures, *r*_*s =*_ 0.6453, 95% CI = 0.5924–0.6927, p<0.001), (Table 2). In addition, there was a moderate agreement between self-reported measures and proportion of daily pills taken by pill count measures, κ = 0.544 (95%CI = 0.4869–0.6011, *p* < 0.001) (Table 3).

**Table 2:** Comparisons between pill count and self-reported adherence levels.

Proportion of daily pills taken by pill count	How often did you take all your pills in the last 3 months? (Self-reported)					
	*None of my pills*	*Very few of my pills*	*About one half of my pills*	*Most of my pills*	*All my pills everyday*	*Total* ^*b*^
0–40%	8 (100.0)	15 (41.7)	8 (30.8)	4 (2.1)	5 (1.9)	40 (7.6)
41–55%	0 (0.0)	12 (33.3)	5 (19.2)	3 (1.6)	2 (0.8)	22 (4.2)
55–69%	0 (0.0)	5 (13.9)	7 (26.9)	20 (10.5)	2 (0.8)	34 (6.5)
70–84%	0 (0.0)	3 (8.3)	5 (19.2)	44 (23.2)	4 (1.5)	56 (10.7)
85–100%	0 (0.0)	1 (2.8)	1 (3.9)	119 (62.6)	251 (95.1)	372 (70.9)
***Total*** ^***a***^	8 (1.5)	36 (6.9)	26 (5.0)	190 (36.3)	264 (50.4)	524 (100)

^a^* total row percentages.

^b*^ total column percentages.

C* Spearman’s **rho = 0.6453.**

**Table 3:** Interrater reliability of Proportion of daily pills taken by pill count vs Self-reported measures.

% Agreement	Kappa	Weighted Kappa	95%CI	P-value
61.45%	0.354	0.544	0.487–0.601	< .0001

Overall (Table 4), 71% (n = 372) of women were estimated to have protective adherence based on pill count (i.e., a pill count of >85%). The median duration since the last visit was 40 days (IQR 22–63), and the median of number of pills taken was 31 (IQR 19–54). Protective adherence was associated with being aged 35 years or older (OR = 2.54, 95% CI = 1.39–4.67, p = 0.002), being divorced/separated/widowed (72.6% vs 56.6%, OR = 2.29, 95% CI = 1.48–3.53, p<0.001), living in a fishing community (62.1% vs 37.9%, OR = 2.13, 95% CI = 1.45–3.13, p<0.001), intending to continue taking PrEP in next 12 months (96.5% vs 88.8%, OR = 0.28, 95% CI = 0.13–0.60, p = 0.001) and having an STI in last 3 months (59.9 vs 42.1, OR = 2.05, 95% CI = 1.4–3.01), p<0.001).

**Table 4:** Factors Associated with Protection Adherence* (n = 372) using logistic regression, bivariate and multivariable predictors of complete adherence (pill count > = 85%).

	Non-protective adherence (n = 152)	Protective adherence (n = 372)	OR (95% CI)	p-value	aOR (95% CI)	p-value
Age, years						
18–24	62 (40.8)	100 (26.9)	Ref.		Ref	
25–34	72 (47.4)	198 (53.2)	1.71 (1.12–2.59)	0.012	1.44 (0.87–2.35)	0.148
35+	18 (11.8)	74 (19.9)	2.54 (1.39–4.67)	0.002	2. 40 (1.17–4.86)	0.017
Education	81 (53.3)	230 (61.8)				
Primary or less	81 (53.3)	230 (61.8)	Ref			
Secondary or high	71 (46.7)	142 (38.2)	0.70 (0.48–1.03)	0.072	0.82 (0.54–1.27)	0.388
Marital status:						
Single/never married	51 (33.5)	70 (18.8)	Ref		Ref	
Married	15 (9.9)	32 (8.6)	1.55 (0.76–3.16)	0.225	1.45 (0.62–3.38)	0.391
Divorced/separated/widowed	86 (56.6)	270 (72.6)	2.29 (1.48–3.53)	<0.001	1.69 (0.98–2.89)	0.055
Location						
Trans Africa Highway	86 (56.6)	141 (37.9)	Ref		Ref	
Fishing community	66 (43.4)	231 (62.1)	2.13 (1.45–3.13)	<0.001	1.45 (0.93–2.25)	0.099
Source of Income						
Sex work only	65 (42.8)	176 (47.3)	Ref			
Sex work and any other occupation	87 (57.2)	196 (52.7)	0.83 (0.56–1.21)	0.344		
Money paid per client (UGX)						
≤5000 (1.4USD)	6 (3.9)	8 (2.2)	Ref			
5000–10000 (1.4 USD– 2.8 USD)	85 (55.9)	211 (56.7)	1.86 (0.62–5.53)	0.263		
≥10000 (≥ 2.8 USD)	61 (40.1)	153 (41.1)	1.88 (0.62–5.65)	0.260		
Sex with more than or equal to 100 men	118 (77.6)	327 (87.9)	2.09 (1.28–3.43)	0.003	2.56 (1.37–4.73)	0.003
Intended to continue taking PrEP in next 12 months	135 (88.8)	359 (96.5)	0.28 (0.13–0.60)	0.001	0.56 (0.24–1.33)	0.193
Preferences in taking PrEP						
Everyday	33 (21.7)	191 (51.3)	Ref		Ref	
Only when I feel I am at high risk	90 (59.2)	112 (30.1)	0.22 (0.14–0.34)	<0.001	0.26 (0.15–0.43)	<0.001
Other	29 (19.1)	69 (18.6)	0.41 (0.23–0.73)	0.002	0.44 (0.23–0.81)	0.010
Condom use in last 3 months with husband						
Always	7 (8.4)	10 (5.6)	Ref.			
Sometimes	25 (30.1)	76 (42.5)	2.13 (0.73–6.19)	0.166		
Never	51 (61.5)	93 (51.9)	1.28 (0.46–3.52)	0.641		
Condom use in last 3 months with regular paying partners						
Always	30 (25.4)	77 (26.9)	(Ref)			
Sometimes	78 (66.1)	176 (61.5)	0.88 (0.53–1.44)	0.613		
Never	10 (8.5)	33 (11.5)	1.29 (0.56–2.93)	0.550		
Condom use in last 3 months with casual partners						
Always	45 (31.3)	131 (35.8)	Ref.			
Sometimes	94 (65.3)	209 (57.1)	0.76 (0.50–1.15)	0.206		
Never	5 (3.5)	26 (7.1)	1.79 (0.64–4.93)	0.263		
Had at least one STI in last 3 months?						
Yes	88 (57.9)	149 (40.1)	Ref.		Ref	
No	64 (42.1))	223 (59.9)	2.05 (1.4–3.01)	<0.001	1.64 (1.07–2.49)	0.020
Depression (PHQ-9)						
Low depression (≤ 10)	79 (51.9)	217 (58.3)	Ref			
High depression (≥ 10)	73 (48.0)	155 (41.7)	0.77 (0.52–1.12)	0.184		

Significant values (p<0.05).

In multivariable analysis (Table 4), protective adherence was independently associated with being aged 35 years or older (aOR = 2 2.40, 95% CI = 1.17–4.86, p = 0.017), being divorced/separated/widowed (aOR = 1.69, 95% CI = 0.98–2.89, p = 0.055), having sex with more than 100 men in the past 3 months (aOR = 2.56, 95% CI = 1.37–4.73, p = 0.003), preferring to take PrEP only when they felt at high risk (aOR = 0.26, 95% CI = 0.15–0.43, p<0.001), living in the fishing community (OR = 1.45, 95% CI = 0.93–2.25, p = 0.099) and having at least one STI in last 3 months (OR = 1.64, 95% CI = 1.07–2.49, Location of residence, marital status, and intention to continue taking PrEP in the next 12 months were not independently associated with protective adherence in the multivariable model.

## Discussion

In this study of Ugandan FSWs using PrEP, almost three quarters (71%) have high protective adherence to PrEP as measured by pill count, and 50.4% by self-reported measures in the last 3 months. Predictors of protective adherence included being older than 35 years, being widowed, divorced, and separated, living in a fishing village and preferring regular rather than risk-based PrEP use. There was both a strong correlation and a moderate agreement between pill count and self-reported adherence.

High protective adherence levels have been shown in previous studies in Sub-Saharan Africa. In the TAPS study, conducted among FSWs in South Africa, self-reported adherence rates ranged from 70 to 85% [14], while a recent study conducted among FSWs in Kampala, Uganda showed adherence rates of 71% [38]. Other studies have shown PrEP discontinuation within six months of initiation [12,27,39–41]) due to reasons such as concerns about acute side effects, perceived low HIV risk and problems associated with daily dosing (pill burden). In this study, we examined adherence in FSWs who had been on PrEP for at least 5 months and were receiving PrEP routine care. In this context it is not surprising that our reported adherence was much higher than many other reports elsewhere [42–44].

Predictors of protective adherence in our study included being older (aged over 35 years), which was also identified in other Sub-Saharan studies [19,20,45]. This age-related trend may be explained by the hope to increase in life expectancy when engaging in a high-risk occupation like sex work as women as seen in another Ugandan study [46]. Older female sex workers may also have had more exposure to adherence messages over time, and thus better understand the benefits of taking PrEP. This has been reported in a study among FSWs in Senegal [47]. In addition, risk-taking behaviours tend to decrease with older age compared with younger women (>25years) [14,48,49] which could explain higher protective adherences among older women in this study. Women from fishing communities had been on PrEP significantly longer than women in Trans African towns (52.5% vs 33.4) and living in fishing communities strongly correlated with protective adherence. Fishing communities have become an important population in planning informed prevention messaging. The increased HIV prevention efforts by key stakeholders and concentrated HIV prevention activities in the fishing communities compared with other areas may be reflected to the higher levels of PrEP adherence in these areas [50].

We observed a strong correlation between pill count and self-reported adherence. This is similar to other studies in Sub-Saharan Africa that have shown moderate to high correlation, including the Partners PrEP study [51], and a serodiscordant couples’ study in East Africa [33]. In addition, there was a moderate agreement between the two methods which means one method can be a substitute for another when measuring adherence or both methods can be used concurrently in making decisions. These results are comparable to other studies that that have found a moderate to high agreement between self-reported and pill count measures [52,53]. It is also important to note both measures are susceptible to bias e.g., social desirability and reporting bias and recall errors. For example, individuals may remove pills or participants may self-report high pill taking behaviours, to appear more adherent than they really are [54,55]. Further validation of these methods is needed to guide selection in low-income settings.

We also found that those who preferred to take PrEP only when at risk were less likely to report protective adherence. This is consistent with previous work indicating that non-daily PrEP dosing may lead to lower adherence compared with daily PrEP use [56]. Taking PrEP only when at risk maybe a better option for those struggling with daily dosing as it fosters a sense of self control and self-efficacy [57,58]. However, understanding and determining “being at risk” depends on the user’s ability to understand and to predict their risk of HIV acquisition and take pill in a manner that protects that risk episode.

Being widowed, divorced and separated rather than having a regular partner were independently associated with protective adherence to PrEP. This could possibly be due to less worry and anxiety, as women with sexual partners may be concerned their sexual partners will find out they are taking PrEP [59,60]. Further research exploring the role of personal and social relationship factors on PrEP adherence is important as it could better support interventions to improve PrEP adherence. This study also shows women who did not have an STI in the last 3 months had higher levels of protective adherence than those who had had an STI. This could be attributed to better condom use at last sex among women in this study with regular paying partners (53.7% vs 46.3%) and casual partners (59.2% vs 40.8%).

We acknowledge that self-reported measures and pill count may not be accurate measures of adherence compared to biological methods, as seen in other Sub-Saharan studies [43,44,52]. However, in some Sub-Saharan African studies, self-reported measures have correlated well with plasma drug concentrations. In a Botswanan oral PrEP efficacy trial [61], a modest correlation was found between self-report and TDF/FTC drug levels (0.28). The clients who self-reported 100% adherence had higher drug concentrations in their system. There was also substantially higher levels of detectable TFV (Tenofovir) and FTC (emtricitabine) drug in those with pill counts ≥90% compared with those with pill counts of <90% (61). The iPrEX study also showed self-report and pill count were significantly associated with higher drug levels [62] while another clinical trial among discordant couples in East Africa found high levels of adherence based on clinic-based pill counts and tenofovir drug levels [51]. These findings provide support for the validity of self-report measures in this study. In addition, in the absence of biological data on adherence, which is the situation for most clinical and PrEP programs in low-income settings, self-reported measures can be an important marker of protective adherence. The most objective methods such as drug concentrations in biological specimens are expensive and are not feasible in many of these low-income settings. Instead, inexpensive, simple methods like self-report, although subjective and susceptible to bias, may be an option. Future research should investigate the incidence of HIV among this population to confirm these findings, as protective adherence is associated with low HIV incidence.

Our study has a number of limitations. First, it was not able to confirm adherence by drug level testing, but as discussed above, self-reported measures have been proven credible in some settings [61,63]. Second, all our variables relied on self-report which may be influenced by social expectancies and recall bias. Thirdly, our approach to pill count was limited by potential differences in time between refill visits. Women’s pills were counted based on the pills dispensed at last visit compared with the numbers of pills returned at the last date of interview. The time period for the pill counts (median 40 days) was different to the three months used for the self-report measure. However, other studies in Africa have used the same method of pill count whose results have been reliable [63–65]. Fourthly, although our participants were similar in risk behaviours to other samples of FSWs in Uganda, they are unlikely to be representative of all FSWs living in Uganda. Our sample mainly focused on rural FSWs taking PrEP and attached to specific PrEP hubs/clinics. Despite these limitations, our study is the first study of PrEP adherence in the context of FSWs living in two high risk areas in Southwestern Uganda fishing communities and towns along the Trans-African Highway.

## Conclusion

The findings from this study indicate that a high proportion of FSWs in Southwestern Uganda prescribed PrEP had protective levels of adherence, based on pill count and self-reported measures. Though self-report measures may provide valuable information, additional targeted research on adherence measures using drug concentrations in biological specimens maybe more reliable. Larger studies that definitively assess adherence by measuring HIV incidence may help to assess further the effectiveness of PrEP especially where more objective measures may not be possible. Despite the high adherence levels in our study, there is need for key stakeholders to strengthen other alternative HIV prevention measures like condom use, especially when PrEP users have perio1ds of discontinuation. As we continue to promote PrEP and advocate for its access, it’s important to intensify further STI screening, testing, and treatment among such high-risk populations. Long-acting injectable cabotegravir has proven to be safe and effective in preventing HIV in cisgender women [66], this could be another promising option for HIV prevention in such high-risk populations.

## Supporting information

S1 File (DOCX)
